# Crystal structures of the human Dysferlin inner DysF domain

**DOI:** 10.1186/1472-6807-14-3

**Published:** 2014-01-17

**Authors:** Altin Sula, Ambrose R Cole, Corin Yeats, Christine Orengo, Nicholas H Keep

**Affiliations:** 1 Crystallography, Biological Sciences, Institute for Structural and Molecular Biology, Birkbeck University of London, Malet Street, London WC1E 7HX, UK; 2 Structural & Molecular Biology, Darwin Building, Institute for Structural and Molecular Biology , University College London, Gower Street, London WC1E 6BT, UK; 3 Current Address: Infectious Disease Epidemiology, Imperial College London, St Mary’s Campus, Norfolk Place, London W2 1PG, UK

**Keywords:** Dysferlin, Limb girdle muscular dystrophy 2B, Arginine-tryptophan stacking, DysF domain, Crystal structure

## Abstract

**Background:**

Mutations in dysferlin, the first protein linked with the cell membrane repair mechanism, causes a group of muscular dystrophies called dysferlinopathies. Dysferlin is a type two-anchored membrane protein, with a single C terminal trans-membrane helix, and most of the protein lying in cytoplasm. Dysferlin contains several C2 domains and two DysF domains which are nested one inside the other. Many pathogenic point mutations fall in the DysF domain region.

**Results:**

We describe the crystal structure of the human dysferlin inner DysF domain with a resolution of 1.9 Ångstroms. Most of the pathogenic mutations are part of aromatic/arginine stacks that hold the domain in a folded conformation. The high resolution of the structure show that these interactions are a mixture of parallel ring/guanadinium stacking, perpendicular H bond stacking and aliphatic chain packing.

**Conclusions:**

The high resolution structure of the Dysferlin DysF domain gives a template on which to interpret in detail the pathogenic mutations that lead to disease.

## Background

Dysferlinopathies are a group of autosomal recessive inherited late onset progressive muscular dystrophies caused by malfunction of dysferlin protein. Mutations in the dysferlin protein cause three phenotypes called limb girdle muscular dystrophy type 2B [1], Miyoshi myopathy [2], and distal anterior compartment myopathy [3]. Dysferlin is not a part of the dystrophin-glycoprotein complex, but its function is linked with calcium-activated membrane repair caused by fusing aggregated intracellular vesicles with the sarcolemma at the site of injury [4-6]. The mechanism of membrane repair is not yet determined in detail and the specific role of dysferlin needs to be defined at the structural level.

Dysferlin, a member of the ferlin protein family, is a type II anchored membrane protein with a single C terminal helix buried in the membrane. Ferlin proteins are defined as containing four or more C2 domains and a C terminal trans-membrane helix. There are 6 ferlin proteins expressed in human; dysferlin, myoferlin, otoferlin, Fer1L4, Fer1L5, and Fer1L6 [7]. Myoferlin is the most similar paralogue to dysferlin. Both proteins are predicted to have the same domain composition with overall sequence identity of 56%. Dysferlin is expressed in most tissues but is found in abundance in skeletal muscle, heart, brain and placenta.

**Figure 1:**
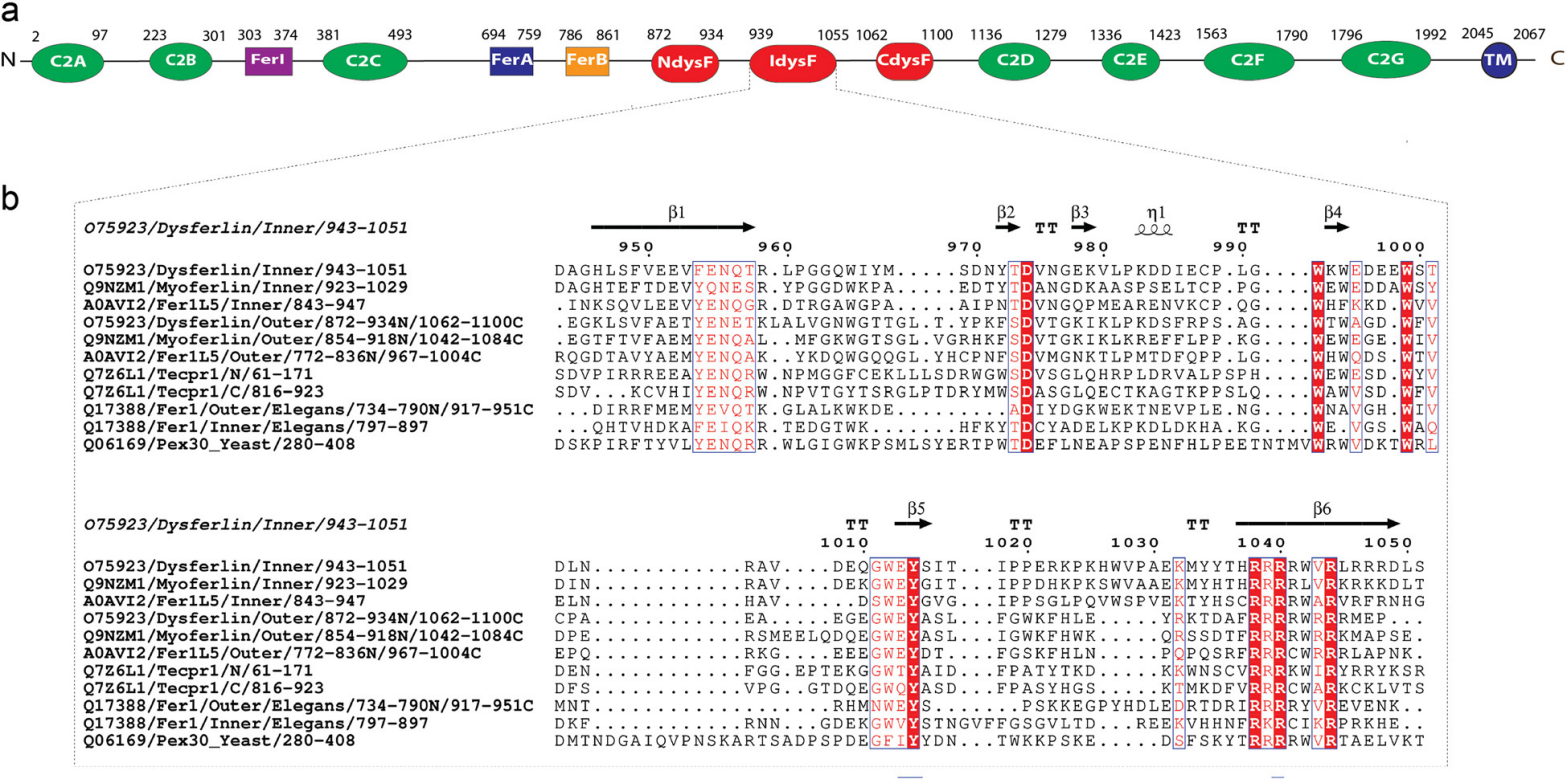
**Domain organisation of dysferlin and alignment of DysF domains. a)** Multiple domain architecture of human dyferlin protein (Gene3D and pFam). **b)** Multiple sequence alignment of inner and outer DysF domain of Human Myoferlin, Dysferlin, Fer1l5; human Tecpr1 DysF domains; inner and outer DysF domain of *C. elegans* FerI protein, and Yeast Pex30 DysF domain (aligned with mafft and drawn with ESPript).

The multiple domain architecture of dysferlin was analysed by a combination of Gene3D [8], SMART [9] and Pfam [10] domain family resources. The domain architecture is predicted to consist of seven C2 domains (C2A to C2G), three Fer domains (FerA, FerB and FerI), two DysF domains, one nested inside the other, and a C terminal trans-membrane domain (Figure 1a). C2 domains are found in hundreds of proteins and many are known to bind to phospholipids or proteins, often in a calcium dependent manner [6]. In dysferlin and myoferlin C2A binds to phospholipids in a calcium dependent manner. [11,12]. The other C2 domains do not show calcium-dependent binding to lipids, but do show some calcium independent binding to phospholipids [11,12]. The other C2 domains are believed to interact with dysferlin binding proteins or to be involved in dimerisation [13]. Recently it has been shown that there is a minor variant of the dysferlin C2A domain, C2Av1, that does not bind via calcium [14]. Crystal structures of the canonical and variant structures of C2A show conservation of structure, even where the sequence is not conserved. Biophysical characterisation of the interactions with phospholipids and calcium indicate that the C2A and C2Av1 domains are highly conformationally flexible [14]. The Fer domains (FerA, FerB, and FerI) are short conserved regions found only in the ferlin protein family and are not yet shown to be folded domains.

Dysferlinopathy causing mutations are dispersed throughout the length of the protein, but many fall in the DysF domains [15,16]. One DysF domain is inserted into the other DysF domain, by gene duplication, forming an inner DysF domain and a two part (N terminal and C terminal) outer DysF domain [17]. The function of the DysF domain is unknown. The human myoferlin (dysferlin paralogue) inner DysF domain structure was solved by Nuclear Magenetic Resonance (NMR) and showed a novel fold. This consists of two long beta strands connected by a long loop that caps the sheet edges in certain sections [18]. The structure contains arginine/tryptophan stacks that holds the fold together and are largely conserved throughout DysF domain sequences (Figure 1b).

In this study, we have determined the three dimensional structure of human dysferlin inner DysF domain by X-ray crystallography at 1.9 Å. This is the first DysF domain crystal structure.

## Results and discussion

### Structure characterisation

**Figure 2:**
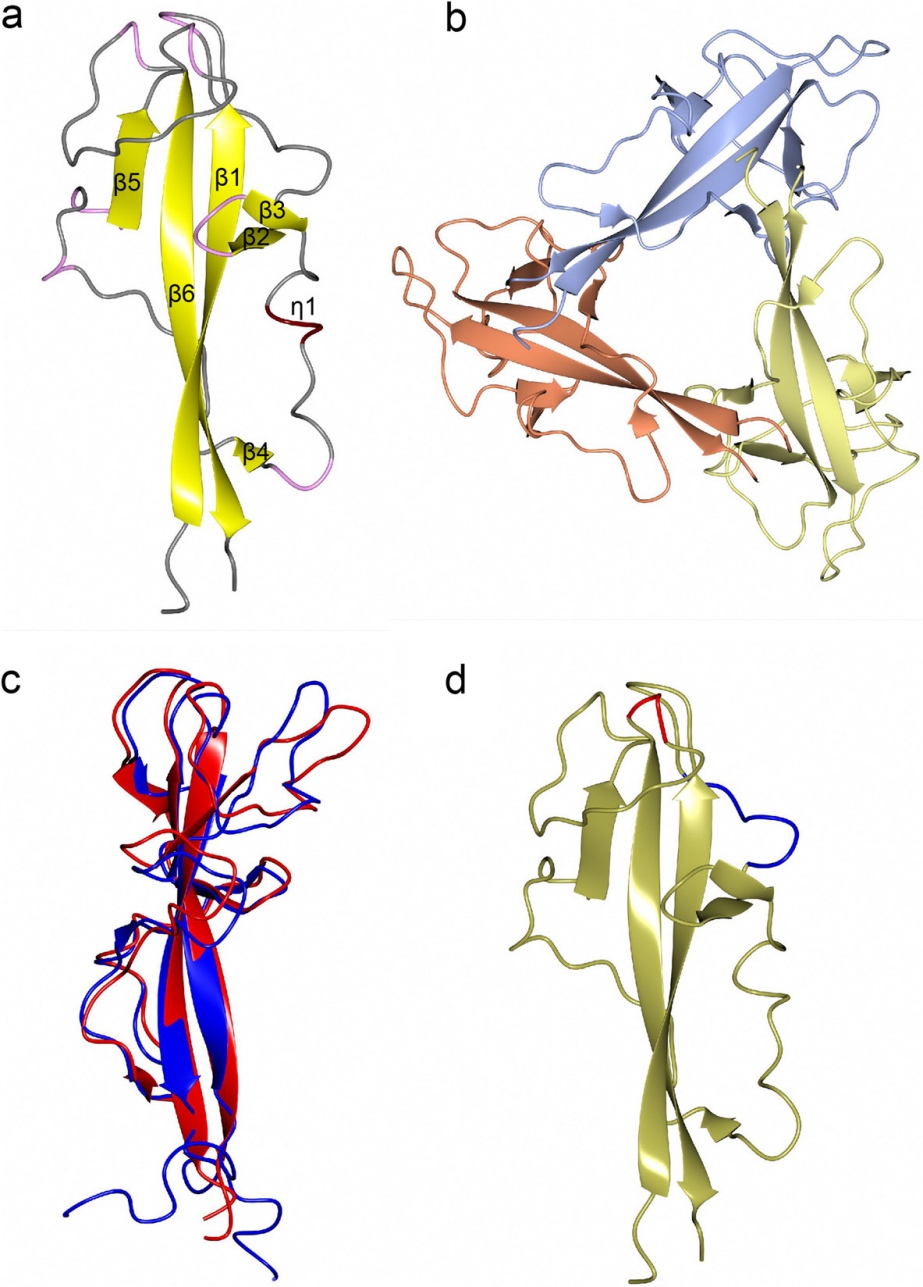
**Comparison of DysF domain structures.** Ribbon presentation of **a)** P2_1_3 asymmetric unit (the beta strands are coloured in yellow, 3 turn in pink, 4 turn in tan and no structure in grey) and **b)** P2_1_2_1_2_1_ asymmetric unit coloured by chain. **c)** Superimposed models of myoferlin NMR DysF domain (blue) with dysferlin crystal DysF domain (red). **d)** Two areas of very high B factor in dysferlin DysD domain coloured in blue (965-971) and red (1018-1021). Orientation as in Figure 2a). Figure drawn with CCP4mg [19].

The structure was solved by molecular replacement with the NMR structure of the inner DysF of myoferlin [18]. The sequence identity between the inner DysF domain in dysferlin and myoferlin is 61% (Figure 1b). Most datasets that were collected, processed and refined in space group P2_1_3 (cubic), with the best resolution dataset diffracting to 1.9 Å. A single dataset was collected in a different space group P2_1_2_1_2_1_ (orthorhombic) to 2.2 Å. The crystal packing is almost the same in the two forms, but the distortions of the perfect cube in the orthorhombic structure means the monomers of the crystallographic trimer in the cubic crystals are no longer identical (Figure 2a, b). This leads to some of the flexible regions (eg residues 965-671) being more visible in some chains with the orthorhombic data. Except where stated otherwise the analysis is based on the single chain in the 1.9 Å structure. Statistics for both datasets are summarised in Table 1 and have been deposited in the Protein Data Bank [PDB:4CAH][PDB;4CAI].

**Table 1:** Data collection and refinement statistics of the dysferlin inner DysF domain

***Data collection***	**PDB 4CAI**	**PDB 4CAH**
Wavelength (Å)	1.037530	0.97718
Space group	P2_1_2_1_2_1_	P2_1_3
Unit-cell parameters		
a, b, c (Å)	74.5, 77.47, 79.89	75.95, 75.95, 75.95
α, β, γ (^o^)	90, 90, 90	90, 90, 90
Resolution range (Å)	50-2.2 (2.27-2.20)	53.7-1.9 (1.94-1.9)
Total number of observation	78153 (6922)	115092 (7574)
Total number unique	24062 (2084)	11803 (760)
Completeness	99.8 (99.9)	100.0 (100.0)
Multiplicity	3.2 (3.3)	9.8 (10)
<*I/σ(I)>*	12.4 (2.3)	30.5 (3.5)
CC(1/2)	0.976 (0.758)	1.00 (0.847)
R_merge_	0.045 (0.466)	0.044 (0.667)
Solvent content (%)	56.8	53.0
Molecule per ASU	3	1
Wilson B factor (Å^2^)	42.2	31.05
*Refinement*		
Resolution range (Å)	44.57-2.2	53.7-1.9
R_work_	0.1858 (0.2457)	0.1775 (0.1932)
R_free_	0.2271 (0.3102)	0.1908 (0.2283)
Reflection, working	22764	11220
Reflection, free	1206	549
Average B factor	61.0	42.0
Rmsd bond angle	1.358	1.76
Rmsd bond length (Å)	0.011	0.015
*Ramachandran Analysis*		
Preferred region (%)	96.7	95.2
Allowed region (%)	2.4	2.9
Outliers (%)	0.9	1.9

Values in parentheses refer to the highest resolution shell.

R_merge_ = Σ (I - < I>) / Σ < I > .

*R*_work_ = Σ (|*F*_obs_ |- |*F*_calc_|)/ Σ|*F*_obs_| for 95% of data. Rfree is the same equation for 5% of the data excluded from refinement.

The inner DysF domain construct starts at residue Met942 and ends at Gln1052 and has a Ser on the N terminus from the TEV protease site. The additional Ser, Met942 and Gln1052 are not visible in the structures. The main secondary structure consists of two long antiparallel β-strands, one at each terminus (N terminus 946-958, C terminus 1036-1049). These β-strands are connected with a long loop (77 residues). The loop caps the edge of the N terminal β-strand with main chain hydrogen bonds from residues 966, 971 and 973. The C terminal β-strand is capped with main chain hydrogen bonds from residues 993, 995, 996, 1000, 1013 and 1015. These give short β strands within the linking loop. There is also a single turn of 3_10_ helix from 983 to 986 (Figures 1b, 2a). This secondary structure is also conserved in the NMR structure of the myoferlin inner DsyF domain [18].

The structure is highly conserved between independent copies in the crystals. The crystallographic trimer of the P2_1_3 crystal superimposes onto the trimer in the P2_1_2_1_2_1_ asymmetric unit with an RMSD Calpha of 0.89 Å over 326 residues of the trimer. The four individual chains from the two assymetric units superimpose no worse than 0.79 Å Calpha RMSD over 108 residues for any pair. The conservation with the NMR structure of myoferlin is also extremely high with a Calpha RMSD of 1.78 Å over 106 residues being the worst and 1.45 Å over 103 residues being the best of the 20 models (Figure 2c). The biggest variation between dysferlin models is between 965 and 971 and 1018 and 1021 (Figure 2d). These are also the regions with the highest temperature (B) factors in dysferlin, the regions with the largest RMS between NMR models in myoferlin, as can be seen from the CING database [20,21], and the regions that differ most between dysferlin and myoferlin. However the region from 958-960 also has high B factors, while conserving the backbone trace between structures quite well.

The trimer (with 9 phosphates) is predicted to be the stable assembly by PDBePISA [22]. Formation of the assembly buries a total 5250 Å^2^. The interface between the protein chains (repeated 3 times in the trimer) forms 5(pisa) - 7(ccp4mg) hydrogen bonds and 3 salt bridges. Three of these are mainchain-mainchain, with β3 forming intermolecular β strand links to β1 as well as the intramolecular H bonds to β6. 2 H bonds are mainchain-sidechain and the others are sidechain-sidechain. Indeed the formation of β3 is probably driven by the trimerisation. The relatively low B factor in this side of the loop may be partially caused by this packing. We have no evidence from gel filtration or NMR spectroscopy (data not shown) for the existence of a trimer, nor is there any likely way to invoke a trimer *in vivo*. There are two DysF domains in dysferlin and the reported oligomeric state of the full length protein is a dimer. Therefore two or four DysF domains seems more likely higher order assemblies, although extending the beta sheet either with other DysF domains or other proteins does seem to be a likely method of interaction.

### Arginine/tryptophan (R/W) stacks

**Figure 3:**
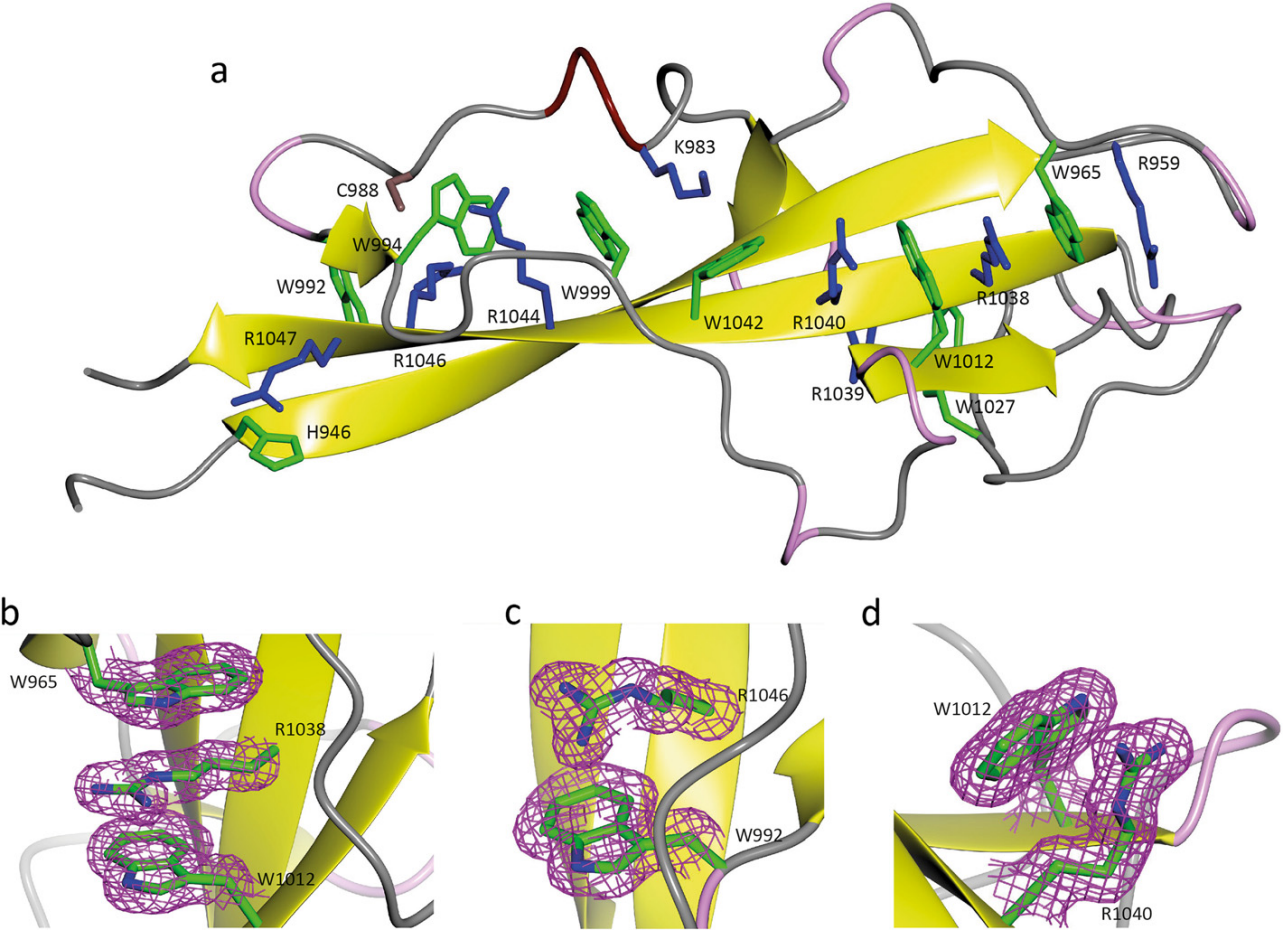
**The aromatic/arginine stack motif. a)** Stick representation of the residues involved in W/R stacks; arginines and lysines are coloured blue, aromatic residues are coloured green, and one cysteine residue in brown. Three forms of stack formation: **b)** parallel (W1012/R1038/W965), **c)** perpendicular (R1046/W992), and **d)** aliphatic stacking (R1040/W1012). Maps are 2mFo-DFc from Phenix.refine contoured at 1.03 Sigma. Figure drawn with CCP4mg [19].

As reported for the myoferlin inner DysF domain [18], the dysferlin DysF domain is held together by arginine/aromatic sidechain stacking. Superficially there is a stack of arginines and tryptophans which runs the entire length of one face of the beta sheet, and a single small group on the other face (Figure 3a). However, at the good resolution of these crystal structures, we can give a much confident description of the exact nature of the interactions. In particular arginines can interact with aromatics either in a stacked (ie with the guanadinium parallel to the aromatic ring- above the six membered ring) [23] or in an H bond (with the amino group pointing at the ring and the guanadinium plane perpendicular to the aromatic ring). Theoretical calculations favour the H bond arrangement in vacuum but the stacked arrangement in water [24]. We do see both these types of interaction but a number of the arginine-guanadinium groups do not lie above the centre of aromatic ring. In fact the atlas of sidechain interactions [25,26], does not have the Arg –Trp pairs above the centre of the ring in most of the clusters. Only one cluster lies above the 6 membered ring. In four of the six cases the guanadiniums lie above or beyond the NE-CZ containing edge of the ring and in one case beyond the opposite edge of the indole. In these cases there is still a hydrophobic interaction of the aliphatic sidechain with the ring and the guanadinium is available to form other H-bonds. A working definition of aliphatic stacking is that at least two side chain atoms are within 3.8 Å of Trp ring atoms (the CH2-CHar Van der Waals radii add up to 3.74 Å [27]).

The details of the R/W interactions are laid out in Table 2 and examples shown in Figure 3. The analysis shows that in fact there is not one continuous stack, but three on one face of the sheet. R1046 is to one side of W994, with only one non-H atom within 3.8 Å. Instead Cys988 forms an aromatic H bond interaction with W994. The second break occurs between, R1040 and W1042, where both side chains hydrogen bond to E955, but there is no direct interaction between the two. On the other face R1039 stacks with both F954 and W1027. Most but not all the stacking interactions are conserved in the myoferlin inner domain, but the NMR structure does not define which mode of packing is taking place. By sequence homology most of these interactions would be found in the dysferlin outer dysf domain (sequence identity 32%). The inner dysf domain of myoferlin lacks equivalents of K983 and W1042, although the overall fold is well conserved; conversely the outer dysferlin dysF domain lacks an equivalent of C988 and R1048, so in both cases the stacks will be a bit shorter.

**Table 2:** Aromatic arginine stack interactions in DysF domains

**Stack**	**Dysferlin inner domain**	**W/R type**	**Interactions**	**Myoferlin inner domain equivalent (Residue mentioned in interaction)**	**Outer Dysferlin DysF equivalent**
1	R1048	Aliphatic W992	Planar interaction to Y1034 in crystal contact	K1029	P1100
			H Bond to Q1010 in crystal contact		
			Interacts with crystallisation phosphate		
			Guanadinium Beyond NH/CZ of W992		
1	W992		H bonds to crystallisation phosphate	W973	W924
1	R1046	Perpendicular W992	NH points to 6 membered ring of W992	R1027	R1096
			H bonds to crystallisation phosphate		
			H bond to E951	(D392)	(A879) ?E880
2	C988		H bond to 5 membered ring of W994	C969	P920
	W994			W975	W926
	R1044	Planar W994/ Aliphatic W999	Planar stack above 5 membered ring W994	R1025	R1096
2	W999		R1044 guanadinium is beyond NE/CZ edge	W980	W930
			Aliphatic interaction with side chain of K983		
2	K983		Just over 4 Å above 6 membered ring of W1042	P964	K915
2	W1042		H bond to E955	L1023 (Q936)	W1094 (E883)
3	R1040	Aliphatic W1012	H bond to E955	R1021 (Q396)	R1092 (E883)
			Guanadinium Beyond NE-CZ of W1012		
3	W1012			W993	W1069
3	R1038	Planar W1012	Planar stack to 5 membered ring of W1012	R1019	R1090
		Planar W965	Planar stack to 5 membered ring of W965		
3	W965		H bond to major conformation of E1031	W946 (E1012)	W894
3	R959	Aliphatic W965	Guanadinum Beyond NE-CZ of W965	R940	K887
			H bonds to major and minor conformations of E1031		
Opposite Face	F954			Y935	Y882
	R1039	Planar F954	Close to planar Stack above F954	R1020	R1090
		Planar W1027	H bond to E1013	(E994)	(E1070)
			Planar Stack above 5 membered ring of W1027 closer to NE than centre		
	W1027			W1008	Unclear

### Dysferlinopathy mutations

**Figure 4:**
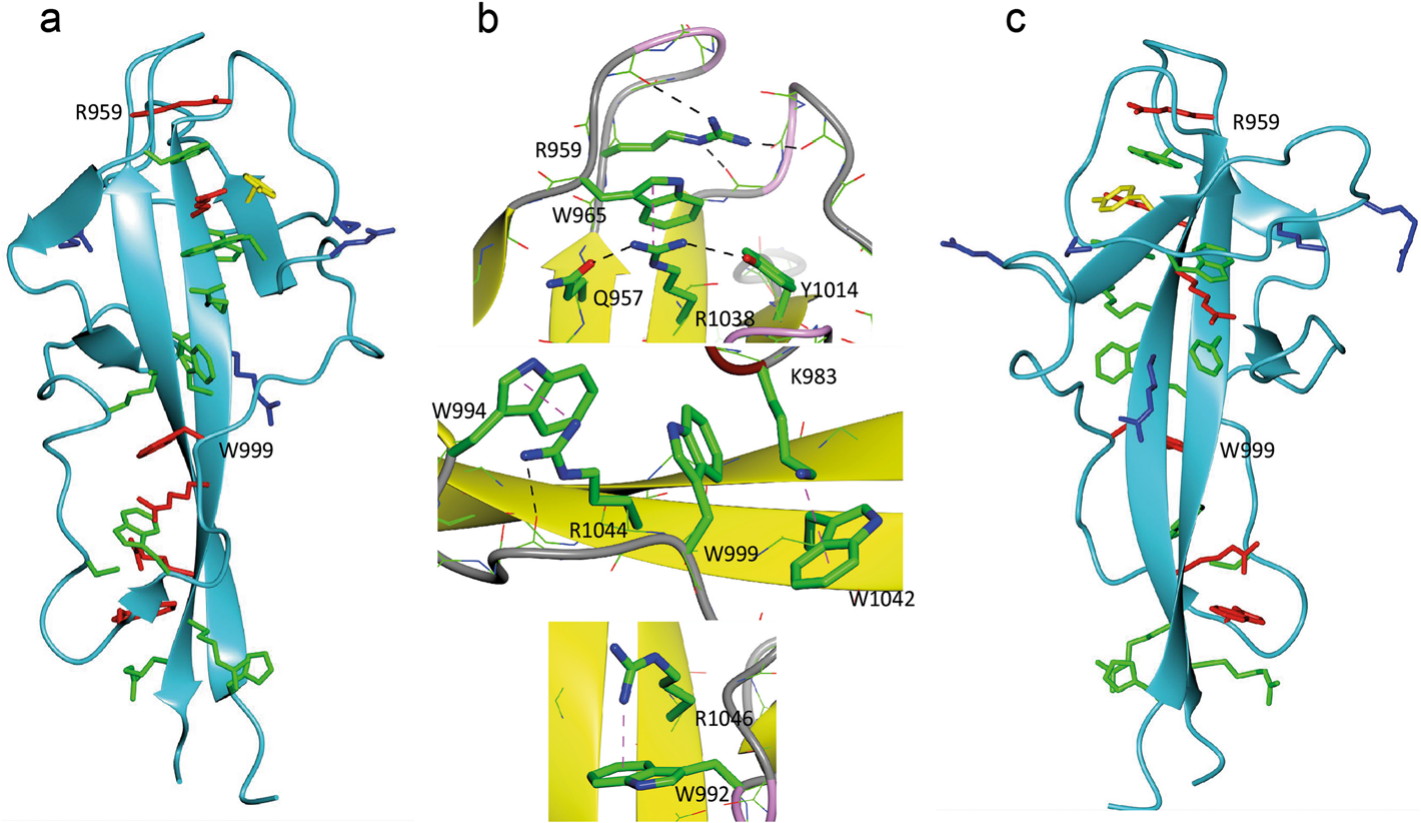
**Missense mutations mapped onto DysF domain structure. (a and c)** Ribbon representation showing W/R stack rotated 180 degrees about the y axis of the page; mutated residues implicated in stack formation (red), mutated residues in the surface (blue), mutated residues stabilising the arginines involved in stack formation (yellow). Other residues of the W/R stack are in green. **(b)**: Interaction and environment of the most common pathogenic mutations (R959, W999 and R1046). H bonds shown as black dotted lines and stacking interactions as magenta dotted lines. Figure drawn with CCP4mg [19].

There are 15 missense mutations in the inner dysferlin domain reported in the Leiden dysferlin mutation database [15,16]. These are summarised in Table 3 which summarises the position. Figure 4 shows position of all the point mutations and close up views of the three most reported mutations. The three most frequent mutations disrupt the R/W stacks and are likely to lead to a less stable or possibly unfolded domain. The unfolded domain may lead to degradation of the entire dysferlin protein. It is also notable that there are several surface residues mutated, although in this very flat domain nearly every residue contributes to the surface, which may indicate that the DysF domain is involved in protein-protein interactions.

**Table 3:** **List of pathogenic missense mutations in the inner dysF domain taken from the Leiden database**[16]

**Residue**	**Disease**	**Number of reports**	**References**	**Role of residue**	**Conservation score**
**Arg959Trp**	LGMD2B & MM	25	[28-34]	In Stack 3	0.522
**Met968Leu**	LGMD2B	1	[35]	In loop. Poorly defined in structure and surface exposed	0.281
**Trp992Arg**	LGMD2B & MM	2	[36]	In Stack 1	1.000
**Trp999Cys**	LGMD2B & MM	24	[35-40]	In Stack 2	1.000
**Glu1009Lys**	?	1	[16]	Surface exposed in loop. Close to phosphate in crystal	0.411
**Gly1011Arg**	DACM	1	[32]	Surface exposed in a loop	0.773
**Tyr1014Cys**	LGMD2B & MM	5	[37,41]	Orientates Arg1038 in Stack 3. H bond OH to guanadinium	1.000
**Arg1022Gln**	LGMD2B	7	[28,37]	Surface exposed. Poor side chain density	0.312
**Pro1029Leu**	MM	2	[42]	Surface exposed	0.062
**Arg1038Gln**	LGMD2B	7	[28,29,42,43]	In stack 3	1.000
**Arg1039Trp**	MM	1	[16]	Opposite face stack	0.923
**Arg1039Leu**	MM	1	[44]	Opposite face stack	0.923
**Arg1041Cys**	MM	2	[31]	Surface exposed. H bond to E952	0.617
**Arg1044Ser**	DAMT	2	[44]	In Stack 2	1.000
**Arg1046His**	MM	11	[42,45,46]	In Stack 1	0.386

LGMD2B- Limb Girdle Muscular Dystrophy 2B. MM-Myoshi Myopathy, DAMT-Distal anterior Myopathy Tibial Onset, Conversation Score was calculated using alignment in Figure 1 and Scorecons program [47].

## Conclusions

The structure of the inner DysF domain of dysferlin confirms the unusual fold of this domain first seen in the myoferlin homologue. The high resolution of the structure allows detailed analysis of the interactions forming the R/W stacking seen in this domain. It also provides a better model for understanding the disease causing point mutations in this domain seen in dysferlin patients. The most common mutations will disrupt the R/W stacking, making the domain more unstable or inherently unfolded, probably enhancing the degradation of the protein. Some of the dysferlin mutants map to the surface, implying that protein-protein interactions may have a role in the function of this domain.

## Methods

### Protein preparation

Human dysferlin cDNA (Jain Foundation) was used to amplify the DNA encoding for the residues 943-1052. The human dysferlin inner DysF domain was cloned into pNic28Bsa4 plasmid (supplied by Dr Opher Gileadi of the Structural Genomics Consortium), which is a modified pET28a plasmid that allows ligation independent cloning [48]. The vector contains an N terminal histidine tag followed by a TEV protease cleavage site. The cloned sequence was confirmed by DNA sequencing and the plasmid transformed into *E. coli* strains Rosetta 2 (DE3) cells. One fresh colony was inoculated into 100 ml of Luria Broth media (LB) and the culture was grown overnight. 8 × 500 ml of LB were inoculated with 1% of overnight culture and incubated at 37°C until the optical density reached 0.5 at a wavelength of 600 nm. Then the cultures were induced with 0.5 mM of IPTG and incubated at 18°C for 20 hours. Cells were collected by centrifuging at 4°C at 4000 rpm for 20 minutes and resuspended in binding buffer (20 mMTris, pH 7.5, 500 mM NaCl, 20 mM imidazole, 10 mM 2-mercaptoethanol). The cells were stored at -20°C. After incubating with EDTA free protease inhibitor cocktail (Roche Applied Science, Switzerland), 0.1% Triton X-100 and DNAseI on ice for 30 minutes, the cells were sonicated at 20 W output for 4 minutes of 4 second on/off pulses for three cycles on ice and then centrifuged at 48,000 g for 1 hour. The protein lysate was applied to a HisTrap (GE Healthcare) affinity column. The column was washed with binding buffer for 10 column volumes and the protein eluted in 20 mM Tris, pH 7.5, 500 mM NaCl, 500 mM Imidazole, 10 mM 2-mercaptoethanol buffer. The eluents containing the dysF domain were pooled together and treated with TEV protease for 20 h at 4°C to remove the histidine tag. Then the volume was decreased to 4 ml by Vivaspin concentrator (Sartorius, Germany) with 5,000 Da molecular weight cutoff. The sample was further purified by size exclusion chromatography using Superdex 200 16/60 column (GE, Healthcare). The protein was purified further by anion exchange chromatography (Resource Q) on a gradient of 0-0.5 M NaCl in 20 mM Tris, pH 7.5, and 5 mM 2-mercaptoethanol. The protein concentration was estimated using NanoDrop spectrophotometer at UV light absorbance at 280 nm (absorbance coefficient = 45950 M^-1^ cm^-1^). The protein sample was concentrated to 10 mg/ml (750 μM) in final buffer (20 mM Tris-HCl, pH 7.5, 120 mM NaCl, and 5 mM 2-mercaptoethanol) and used for crystallisation trials.

### Protein crystallisation and structure determination

The inner DysF domain was screened for crystallisation at 16°C, by the sitting-drop method in 96- well crystallisation plates (Molecular Dimensions), using PACT screen [49]. After 11 days, small crystals appeared in a drop with mother liquor consisting of 0.2 M NaBr and 20% PEG 3350. This crystallisation could not be reproduced initially. 0.2 M NaBr was then added to the protein sample and the crystallisation screen was done again. Bigger crystals were produced in one day, where the reservoir contained 0.04 M potassium dihydrogen phosphate, 16% w/v PEG 8000 and 20% v/v Glycerol. 0.3 mm cubic crystals were grown by the hanging drop method based on this condition. The crystal was cryoprotected in crystallisation buffer with the glycerol concentration increased to 25%. Initial diffraction data was collected to 2.3 Å in house using MicroMax TM -007 rotating anode X-ray generator (λ = 1.54 Å) and Saturn 944+ CCD detector with Varimax optics. Further data was collected at Soleil beamline proxima 1 and at beam ID29 at ESRF. Diffraction images were processed using XDS software package [50], and scaled using aimless in the CCP4 program suite [51]. The initial phases of the dysferlin inner DysF domain were determined by molecular replacement with the program Mr Bump [52] using human myoferlin inner DysF domain [18] [PDB:2K2O] as the search model. The model was manually rebuilt in COOT [53] and refined initially in Refmac [54] and then continued in PHENIX [55]. Data collection and refinement statistics are summarized in Table 1.

No human subjects were directly used in this study. Human mutation data was taken from publicly accessible databases.

## Abbreviations

Å: Ångstrom (10^-10^ m); CING: Common Interface for NMR structure Generation; EDTA: Ethylenediaminetetraacetic acid; IPTG: Isopropyl β-D-1-thiogalactopyranoside; LB: Luria broth; NMR: Nuclear magnetic resonance; RMSD: Root mean square deviation; R/W: Arginine/Tryptophan stacks; TEV: Tobacco etch virus.

## Competing interests

The authors declare that they have no competing interest.

## Authors’ contributions

AS prepared the expression construct and protein, grew the crystals and carried out the refinement and drew most of the figures. AC collected the diffraction data and advised on crystallisation. CY and CO defined the domain boundaries and the multiple domain architecture. NK wrote the first draft of the manuscript. CO and NK devised the project and trained AS. All authors have read and agreed the final manuscript.
